# Supplementary material for: Injured adult motor and sensory axons regenerate into appropriate organotypic domains of neural progenitor grafts
Source: Nat Commun. 2018 Jan 8;9:84. doi: 10.1038/s41467-017-02613-x (PMC5758751; doi:10.1038/s41467-017-02613-x)
Supplement: Supplementary file 1 — Supplementary Information [file 41467_2017_2613_MOESM1_ESM.pdf]

Supplementary Information

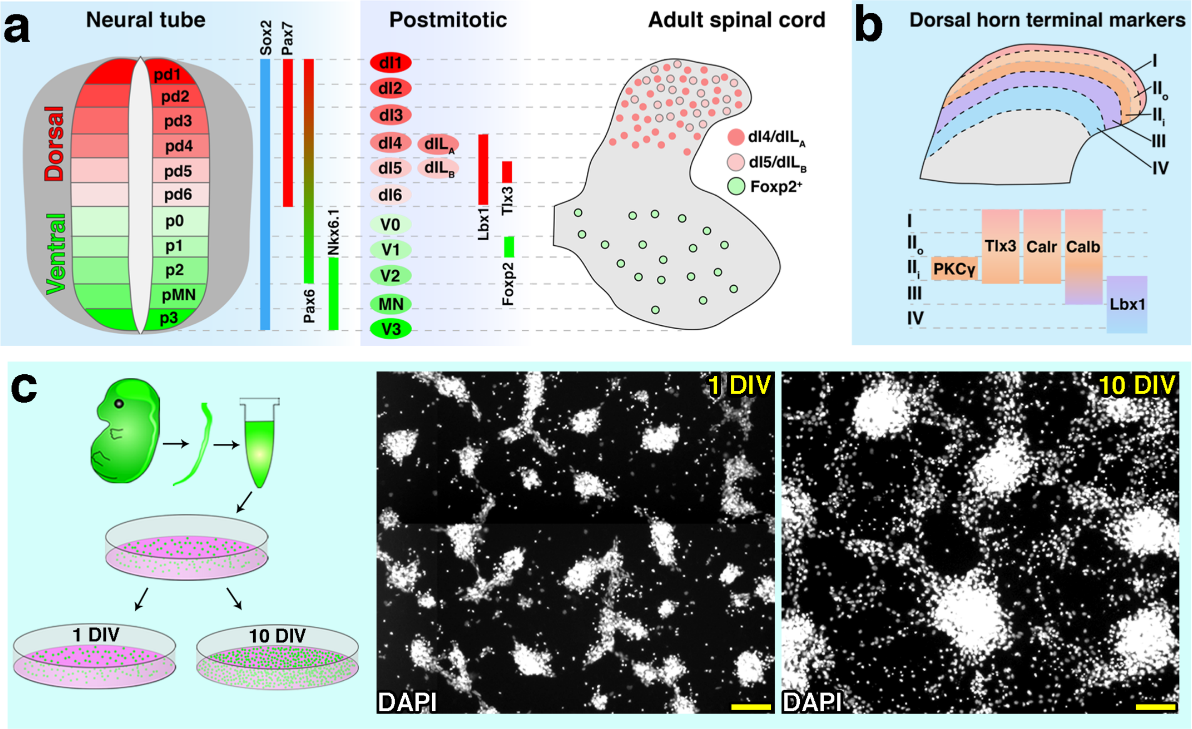

Supplementary Figure 1. ***In vivo*** **spinal cord development and** ***in vitro*** **NPC culture.** (**a**) Cartoon illustrating spinal cord development. **Left:** In the neural tube, combinatorial expression of transcription factors (TFs) defines dorsal (pd1-pd6) and ventral (p0-p3) neuronal progenitor domains. Sox2 is expressed by all neuronal progenitors in the ventricular zone, whereas other TFs are expressed by subsets of dorsal and/or ventral NPCs. For instance, Pax6 is expressed by pd1-p2 progenitors; however, Pax6$^+$/Pax7$^-$ NPCs give rise to ventral (V0-V2) lineages[1,2], whereas only dorsal lineages express Pax7[3]. Nkx6.1 is a marker of ventral progenitors that give rise to motor (MN), V2, and V3 neurons[4]. **Right:** These progenitor domains give rise to thirteen classes of post-mitotic neurons, dI1-V3. Distinct classes of mature neurons can be identified by their expression of lineage-specific TFs; *e.g.*, dI3 and dI5/dIL$_B$ neurons express Tlx3[5], dI4-dI6 neurons express Lbx1[6,7], and V1 neurons express Foxp2[8]). Each class of post-mitotic neuron migrates to specific dorsal/ventral locations and undergoes further specification, giving rise to several subclasses with distinct phenotypes. (**b**) In the adult dorsal horn, different neuronal subpopulations within the superficial laminae can be further identified by their terminal expression of molecular markers. Some markers are expressed by neurons that are narrowly distributed within single laminae; *e.g.*, PKCγ$^+$ neurons exclusively populate inner lamina II (II$_i$)[9]. Other markers [Tlx3, Lbx1, calretinin (Calr), calbindin (Calb)] are expressed by neurons residing

within distinct dorsal/ventral bands spanning multiple laminae. (**c**) **Left:** Cartoon schematic illustrating dissociation of spinal cords from E14 rat embryos and *in vitro* culture of spinal cord progenitors for either 24 hours or 10 days. **Middle:** Multiple small clusters of cells are apparent following 24 hours in culture, suggesting zones of increased proliferation. **Right:** At 10 DIV, larger clusters of cells are evident. Scale bars = 100 μm.

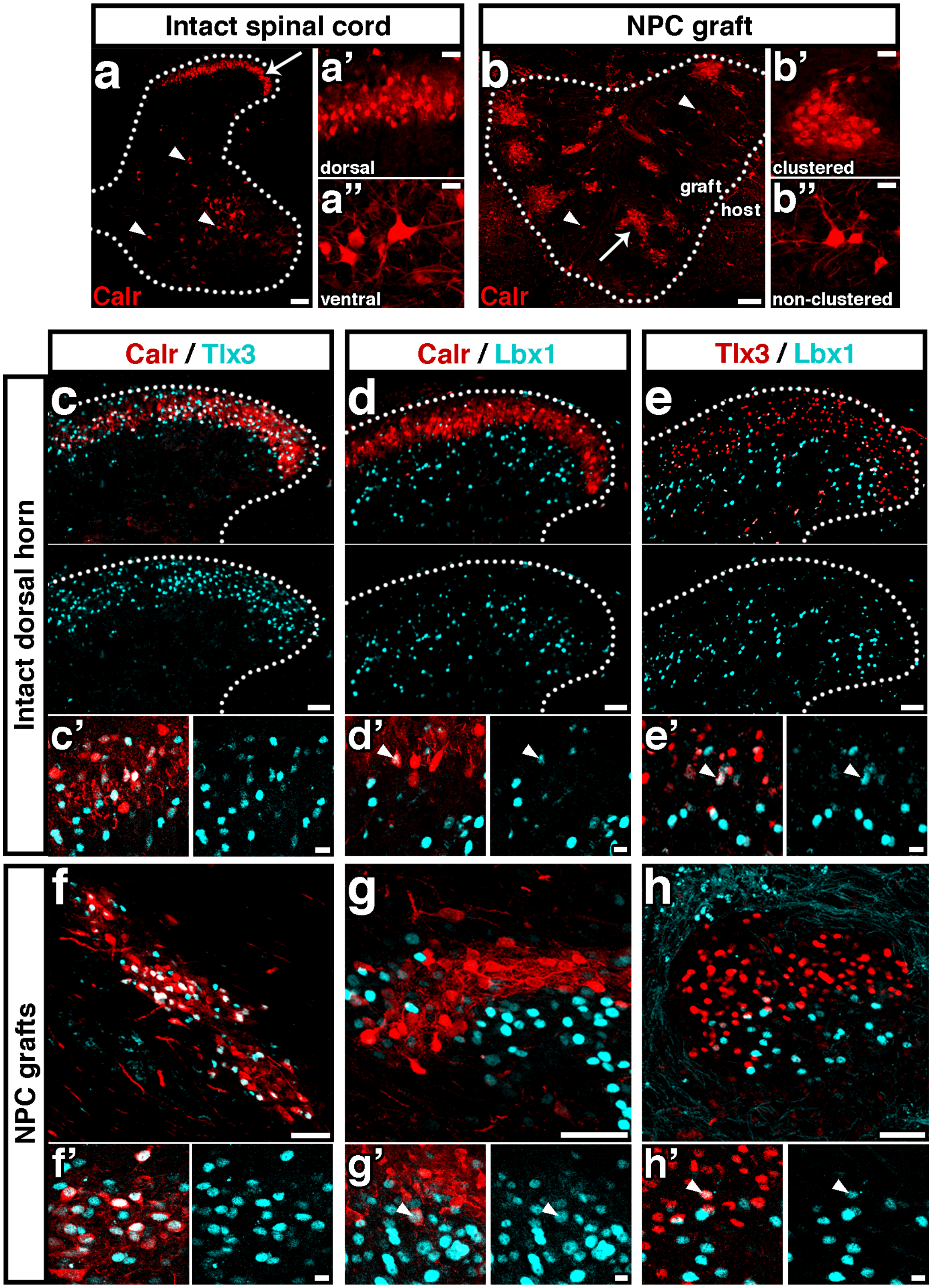

Supplementary Figure 2. Spatial distribution of dorsal spinal cord interneurons in NPC grafts is similar to that of the intact dorsal horn. (**a**) Calretinin (Calr, red) immunoreactivity within the intact cervical spinal cord of intact age P35 rat. Calretinin is expressed in a dense band of clustered, small-diameter neurons in laminae I-II (arrow) and in larger, non-clustered

neurons in the intermediate and ventral gray matter (arrowheads). (**a’, a”**) High-magnification images of calretinin$^+$ neurons (**a’**) in the dorsal horn and (**a”**) in the ventral gray matter. (**b**) Calretinin (red) immunoreactivity in spinal cord NPC graft at six weeks post-transplantation. Arrow indicates cluster of small-diameter calretinin$^+$ neurons and arrowheads indicate larger-diameter, non-clustered calretinin$^+$ neurons in graft. Graft/host boundary indicated with dotted lines. (**b’, b”**) High-magnification images of (**b’**) clustered and (**b”**) non-clustered calretinin$^+$ neurons in NPC graft. (**c-h**) Spatial distribution of dorsal interneuron markers in (**c-e**) the intact cervical spinal cord dorsal horn and (**f-h**) spinal cord NPC grafts. (**c, f**) Calretinin$^+$ (red) neurons and Tlx3$^+$ (cyan) neurons populate the same spatial domains within (**c**) laminae I-II of the intact dorsal horn and (**f**) NPC grafts. (**d, g**) Calretinin$^+$ (red) neuron domains are adjacent to regions populated with Lbx1$^+$ (cyan) neurons within (**d**) the intact dorsal horn and (**g**) NPC grafts. (**e, h**) Tlx3$^+$ (red) neuronal domains are adjacent to Lbx1$^+$ (cyan) neuronal domains within (**e**) the intact dorsal horn and (**h**) NPC grafts. Insets (**c’-h’**) are high-magnification images with arrowheads indicating cells immunoreactive for either (**d’, g’**) calretinin (red) and Lbx1 (cyan) or (**e’, h’**) Tlx3 (red) and Lbx1 (cyan). Scale bars = 100 μm (**a, b**); 50 μm (**c-h**); 25 μm (**a’, a”, b’, b”**); 10 μm (**c’-h’**).

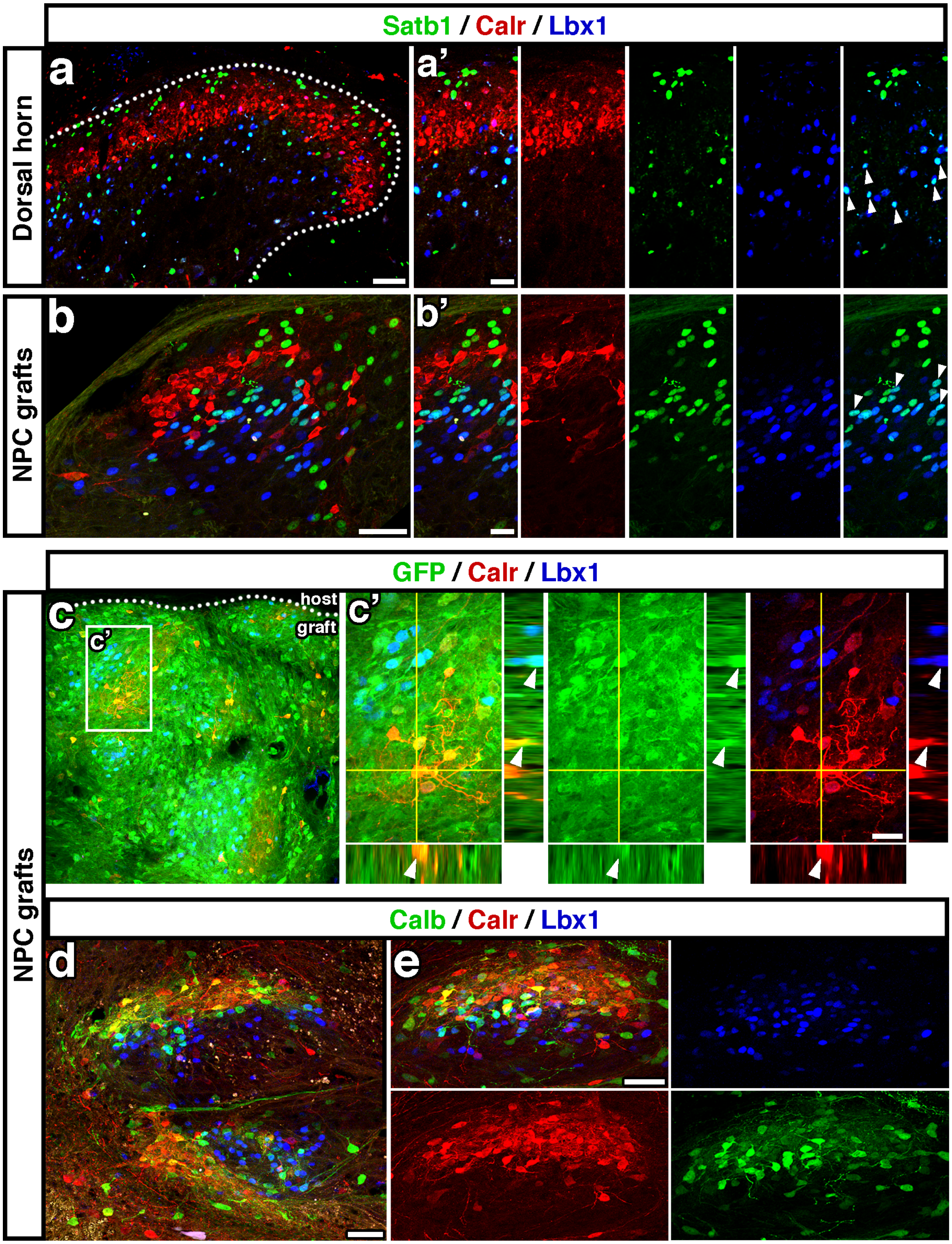

Supplementary Figure 3. Dorsal interneuron domains in NPC grafts exhibit similar cytoarchitecture to the intact dorsal horn. (**a-b**) Laminar distribution of interneurons within (**a**) the intact cervical dorsal horn and (**b**) NPC graft. (**a**) In the intact dorsal horn, calretinin^{+}

(Calr, red) neurons are restricted to laminae I-II, Lbx1$^+$ (blue) neurons reside in lamina III-IV, and Satb1$^+$ (green) neurons are located immediately dorsal and ventral to the band of calretinin$^+$ neurons. Some Satb1$^+$ nuclei located ventral, but not dorsal, to the calretinin$^+$ band are also immunoreactive for Lbx1 (arrowheads). (**b**) Dorsal interneuron cluster in NPC graft, with laminar distribution of Satb1$^+$ (green), calretinin$^+$ (red), and Lbx1$^+$ (blue) neurons. Cells co-expressing Satb1 and Lbx1 (arrowheads) are located on only one side of the calretinin$^+$ neuron band. (**c**) Distribution of calretinin$^+$ (Calr, red) and Lbx1$^+$ (blue) neurons within a GFP$^+$ (green) graft at six weeks post-transplantation. Graft/host boundary indicated with dotted line. (**c'**) Orthogonal view in inset shows GFP expression in dorsal sensory interneurons within grafts (arrowheads). (**d-e**) Typical examples of NPC graft dorsal interneuron clusters, showing laminar distribution of calbindin$^+$ (Calb, green), calretinin$^+$ (red), and Lbx1$^+$ (blue) neurons. Curvature of the laminae I-II neuron (calretinin$^+$ and calbindin$^+$) bands is evident. Scale bars = 50 µm (**a-e**); 25 µm (**a', b', c'**).

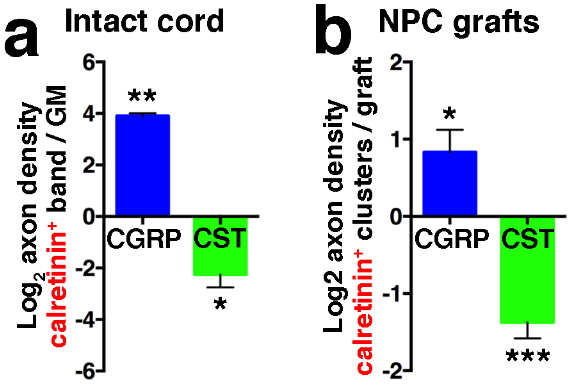

Supplementary Figure 4. Quantification of CGRP^{+} axon enrichment and CST axon avoidance of laminae I-II domains in intact spinal cord and NPC grafts. (**a**) Quantification of CGRP^{+} (blue, n = 3) or CST axon density (green, n = 4) within the laminae I-II calretinin^{+} band in the intact cervical spinal cord. Data is expressed as $\log_2$[axon pixel density in calretinin^{+} band divided by total axon density in spinal cord gray matter (GM)]. Mean ± SEM; *$p < 0.05$, **$p < 0.005$ by single sample t-test with Bonferroni correction for multiple comparisons. (**b**) Quantification of host CGRP^{+} (blue, n = 15) or CST axon density (green, n = 15) within calretinin^{+} neuron clusters in spinal cord NPC grafts at 6 weeks post-transplantation. Data is expressed as $\log_2$(axon pixel density in calretinin^{+} clusters divided by total axon density in grafts). Mean ± SEM; *$p < 0.05$, ***$p < 0.0001$ by single sample t-test with Bonferroni correction for multiple comparisons.

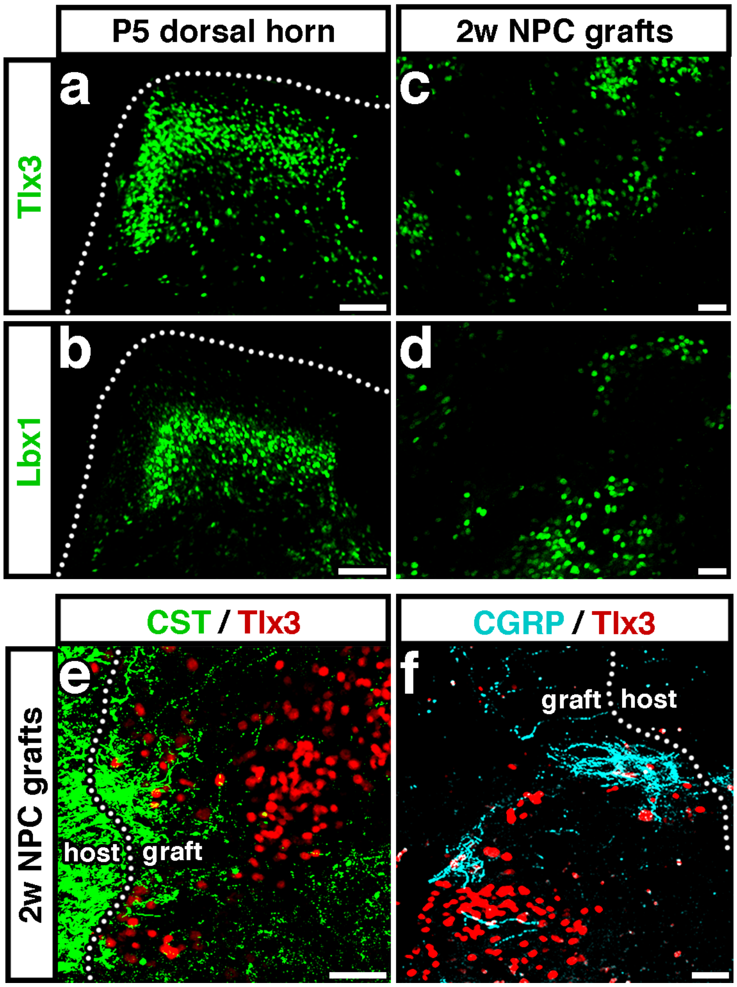

Supplementary Figure 5. Dorsal spinal cord interneuron clusters are present in NPC grafts at 2 weeks post-transplantation. (**a-b**) Transverse images of age P5 rat spinal cords, magnified to show dorsal horns. Expression of (**a**) Tlx3 and (**b**) Lbx1 is largely restricted to bands of cells in the dorsal horn of the spinal cord. (**c-d**) Images from spinal cord NPC grafts at 2 weeks post-transplantation. Clusters of (**c**) Tlx3^{+} and (d) Lbx1^{+} cells are present within graft tissue. (**e**) Image of host CST axons (green) during the initial phase of regeneration into spinal cord NPC graft (2 weeks after injury and grafting). CST axons avoid Tlx3^{+} (red) neuron clusters. (**f**) Host CGRP^{+} axons (cyan) innervate Tlx3^{+} (red) neuron clusters within spinal cord NPC grafts at 2 weeks post-transplantation. Graft/host boundaries in **f** and **g** indicated with dotted lines. Scale bars = 50 µm.

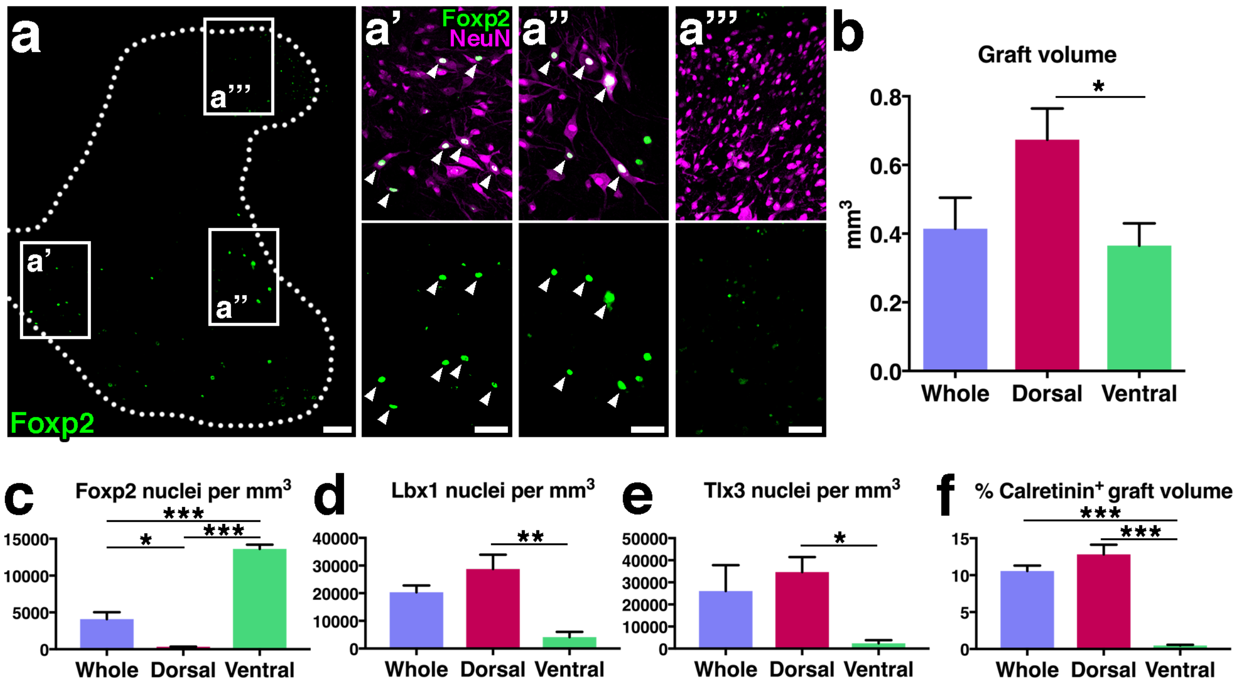

Supplementary Figure 6. Transplantation of regionally-restricted spinal cord NPCs alters graft constituency of dorsal and ventral interneurons. (**a**) Transcription factor Foxp2 (green) is expressed in V1 interneurons in the intact cervical spinal cord of age P35 rats; neurons labeled with NeuN (magenta). Insets show Foxp2$^+$ neurons in the ventral spinal cord (**a**' – **a**"; arrowheads) and an absence of Foxp2$^+$ neurons in dorsal spinal cord gray matter (**a**"'). Scale bars = 100 μm (**a**), 50 μm (**a**' – **a**"'). (**b**) Quantification of graft volume for whole (*n* = 6), dorsal (*n* = 9), or ventral (*n* = 6) grafts. *$p = 0.05$. (**c**) Quantification of Foxp2$^+$ nuclei per mm^3 in whole (*n* = 2), dorsal (*n* = 2), and ventral (*n* = 3) grafts. (**d**) Quantification of Lbx1$^+$ nuclei per mm^3 in whole (*n* = 2), dorsal (*n* = 5), and ventral (*n* = 5) grafts. (**e**) Quantification of Tlx3$^+$ nuclei per mm^3 in whole (*n* = 2), dorsal (*n* = 2), and ventral (*n* = 3) grafts. (**f**) Quantification of percent graft area occupied by calretinin$^+$ clusters in whole (*n* = 4), dorsal (*n* = 4), and ventral (*n* = 3) grafts. All quantifications were performed on E14 spinal cord graft tissue sections at 6 weeks post-transplantation with 30-μm thickness. All data are expressed as mean ± SEM; *$p < 0.05$, **$p < 0.005$, ***$p < 0.001$ by one-way ANOVA followed by Tukey's multiple comparisons test.

Supplementary Table 1. List of primary antibodies used in this study.

Antibody	Manufacturer & Catalog #	RRID	Dilution
Mouse anti-calbindin (D28k)	Swant; #300	AB_10000347	1:1000
Chicken anti-calretinin	EnCor Biotechnology; CPCA-Calretinin	AB_2572241	1:2000
Rabbit anti-calretinin	Millipore; AB5054	AB_11212775	1:1000
Mouse anti-calretinin	Swant; 6B3	AB_10000320	1:1000
Rabbit anti-c-Fos	Santa Cruz Biotechnology; sc-52	AB_2106783	1:500
Mouse anti-CGRP	GeneTex; GTX10987	AB_378002	1:500
Goat anti-CGRP	Abcam; ab36001	AB_725807	1:1000
Rabbit anti-Foxp2	Abcam; ab16046	AB_2107107	1:1000
Chicken anti-GFP	GeneTex; GTX13970	AB_371416	1:1500
Chicken anti-GFP	Abcam; ab13970	AB_300796	1:1500
Guinea pig anti-Lbx1	gift from Drs. Carmen Birchmeier and Thomas Müller	AB_2532144	1:1000
Goat anti-mCherry	Sicgen; AB0040-200	AB_2333092	1:3000
Mouse anti-NeuN (A60)	Millipore; MAB377	AB_2298772	1:500
Guinea pig anti-NeuN	Millipore; ABN90	AB_11205592	
Rabbit anti-Pax6	Biolegend; 901301	AB_2565003	1:100
Mouse anti-Pax7	DSHB; Pax7-c	AB_528428	1:500
Mouse anti-Nkx6.1	DSHB; F55A12-c	AB_528428	1:500
Mouse anti-Satb1 (C-6)	Santa Cruz Biotechnology; sc-376096	AB_10986003	1:500
Rabbit anti-Sox2	Abcam; ab97959	AB_2341193	1:1000
Guinea pig anti-Tlx3	gift from Drs. Carmen Birchmeier and Thomas Müller	AB_2532145	1:1000

SUPPLEMENTARY REFERENCES

1 Ericson, J. *et al.* Pax6 controls progenitor cell identity and neuronal fate in response to graded Shh signaling. *Cell* **90**, 169-180 (1997).

2 Panayiotou, E. *et al.* Pax6 is expressed in subsets of V0 and V2 interneurons in the ventral spinal cord in mice. *Gene Expr Patterns* **13**, 328-334, doi:10.1016/j.gep.2013.06.004 (2013).

3 Lai, H. C., Seal, R. P. & Johnson, J. E. Making sense out of spinal cord somatosensory development. *Development* **143**, 3434-3448, doi:10.1242/dev.139592 (2016).

4 Sander, M. *et al.* Ventral neural patterning by Nkx homeobox genes: Nkx6.1 controls somatic motor neuron and ventral interneuron fates. *Genes & development* **14**, 2134-2139 (2000).

5 Xu, Y. *et al.* Ontogeny of excitatory spinal neurons processing distinct somatic sensory modalities. *J Neurosci* **33**, 14738-14748, doi:10.1523/JNEUROSCI.5512-12.2013 (2013).

6 Gross, M. K., Dottori, M. & Goulding, M. Lbx1 specifies somatosensory association interneurons in the dorsal spinal cord. *Neuron* **34**, 535-549 (2002).

7 Müller, T. *et al.* The homeodomain factor lbx1 distinguishes two major programs of neuronal differentiation in the dorsal spinal cord. *Neuron* **34**, 551-562 (2002).

8 Benito-Gonzalez, A. & Alvarez, F. J. Renshaw cells and Ia inhibitory interneurons are generated at different times from p1 progenitors and differentiate shortly after exiting the cell cycle. *The Journal of neuroscience : the official journal of the Society for Neuroscience* **32**, 1156-1170, doi:10.1523/JNEUROSCI.3630-12.2012 (2012).

9 Spike, R. C., Puskar, Z., Andrew, D. & Todd, A. J. A quantitative and morphological study of projection neurons in lamina I of the rat lumbar spinal cord. *Eur J Neurosci* **18**, 2433-2448 (2003).
